# Protecting yourself at work

**Published:** 2015

**Authors:** Heather Machin

**Affiliations:** Registered Nurse and Consultant: Fred Hollows Foundation NZ, Aukland, New Zealand. hmachin@hollows.org.nz

**Figure:**
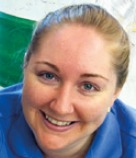
Heather Machin

You need to look after your body when you are at work. This not only prevents you from being injured but it also prevents you from living the remainder of your life with a long-term injury. If you do get injured at work, it may reduce your ability to continue to do that job – which means you might not be able to bring the same amount of money home to support your family. Therefore, anything you can do to protect yourself from injury is important and you must prioritise your health and safety at all times while at work.

Every person has a responsibility to keep themselves and others as safe as possible. Do not take short cuts on safety. Here are some tips on how you can protect yourself, your colleagues, and your patients.

## Know the relevant policies and guidelines

Read your hospital's Workplace (Occupational) Health and Safety policy. This policy outlines the hospital's approach to health and safety in the workplace. Workplace Health and Safety (WHS) is a major component of the risk that hospitals actively manage on a day-to-day basis. This policy is generally supported by other country/regional guidelines or legislation, and accompanied by the hospital's incident reporting system. Additionally, various risk registers and checklists usually accompany the WHS policy.

## Identify potential hazards

It is good practice to develop a risk register where members of the team can write down any potential issues or hazards (e.g. loose cables, or a problem with medication) as they become aware of them.

The next step is to prioritise each issue or hazard so you can deal with the most important ones first. Assess each issue or hazard in terms of:

1. the consequence if this issue or hazard causes harm
2. the likelihood that the issue or hazard will cause harm

Table 1 can help you to prioritise each potential issue or hazard. Look up the consequence (the rows) and the likelihood that it will happen (the columns); where these intersect you can read off how important this particular hazard or issue is and how it should be prioritised.

**Table 1.** Assessing the importance (extreme, high, medium or low) of potential hazards

Consequence of issue or hazard causing harm	Likelihood of potential hazard causing harm				
	Very likely	Likely	Possible	Unlikely	Highly unlikely
Fatality	Extreme	Extreme	Extreme	High	Medium
Major injury	Extreme	Extreme	High	Medium	Medium
Minor injury	High	Extreme	High	Medium	Medium
First Aid	High	Medium	Medium	Low	Low
Negligible	Medium	Medium	Low	Low	Low

- **Extreme and high risk.** It is extremely important to do something about this hazard immediately
- **Medium and low risk.** This hazard may not need your immediate attention, but it should be rectified as soon as it is practical to do so.

## Deal with potential and actual hazards

Once you have prioritised each hazard or issue, work out how to prevent them from causing harm to patients. For example, you can redesign a task to remove an unsafe work practice (e.g. replacing noisy equipment, choosing a less toxic chemical, or providing a trolley for moving heavy files). Here is a list of steps.

- Gather as much information as possible about the potential hazard (do a risk assessment)
  **Figure:**
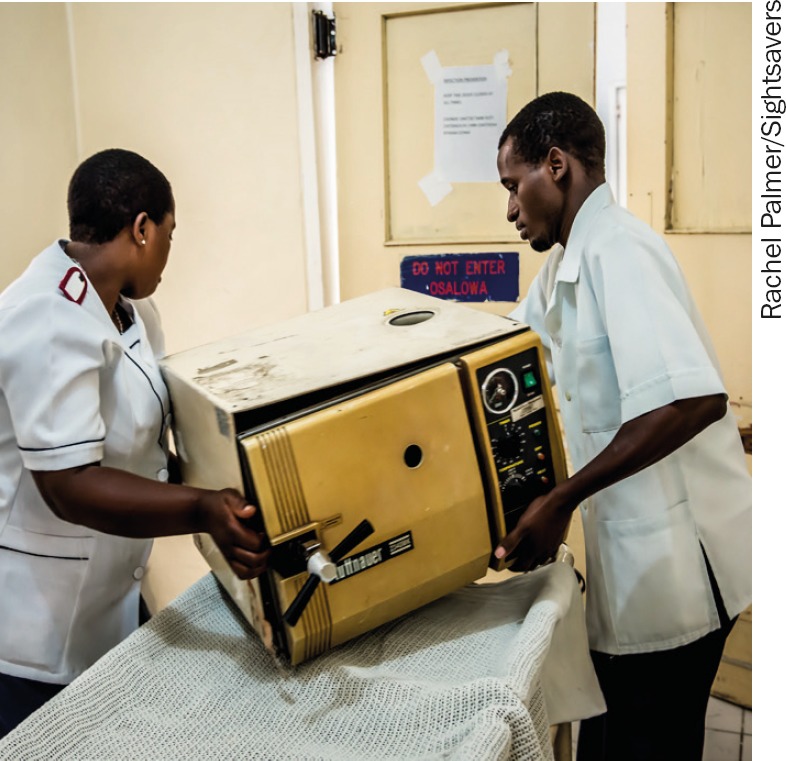
Use correct manual handling techniques to protect your back at work. MALAWI
- Develop and implement a solution to the hazard.
- Look back at the hazard to see if it has been solved or has created another problem (do a review).
- Follow-up after the event – i.e. complete an incident report, talk with your team about what happened, and help to find a way to prevent it in the future. Use the event as an opportunity to educate others.

If something goes wrong:

- Keep yourself safe.
- Rectify the situation if safe to do so within your scope of practice
- Get help if you need it.
- Report it.

**IMPORTANT:** Never place yourself or others in harm's way!

## Look after your work areas

- **Communal areas – i.e. kitchen, meeting rooms, patient waiting areas.** These areas, especially the kitchen, can be dangerous. Staff members must be diligent to prevent and report possible issues. Be careful in this area because both water and electricity are commonly found in the same space – their mix can be dangerous.
- **Maintenance areas – i.e. gas cylinder areas, cleaner's cupboard, biomedical workshops and boiler rooms.** Be extremely careful in these areas. Only enter with permission from the maintenance team. Heat, chemicals, electricity and water may all be hazards in these areas. A strict ‘approved-authorisation-only’ policy should be enforced.
- **In the office.** Look after computer electrical cables and make sure that computers and other electrical equipment cannot overheat. Be careful of tripping over items on the floor too. Have chairs and desks at the correct height to prevent back injury.
  **Figure:**
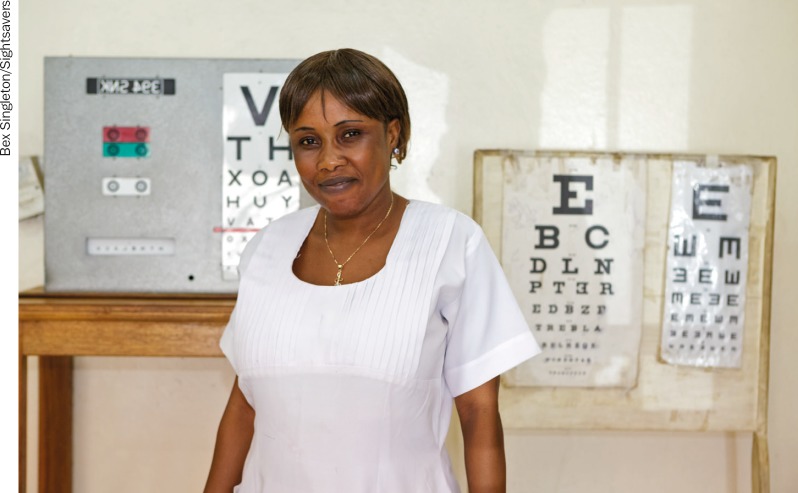
You must prioritise your own health and safety at all times when you are at work. SIERRA LEONE
- **In the clinic.** Clear the pathways for patients. Make sure equipment and stock cannot be tampered with. Have hand wash and gloves available in each room. Clean equipment between each patient, and clean the whole room at the end of the day. Follow Laser Safety policies when using lasers.
- **In the operating theatre.** As in the clinic, ensure that the area is clean and tidy. Extra care needs to be taken in this area because there are often more machines (meaning more electrical cables), which pose a tripping hazard. Also make sure staff members are trained in correct manual handling to prevent injury when transferring patients and moving beds.

## Wear personal protective equipment (PPE)

Personal protective equipment (PPE) is really important. It protects you and your patient. This includes wearing of masks, aprons, gloves, suitable shoes (which cover your toes and provide heel support) and other items such as lead aprons if you are working in the radiology department. Other important key items are laser safety goggles for when you are using a laser.

Be aware that, when you wear gloves, you still need to wash your hands before and after. Buy non-latex gloves whenever possible. Whereas latex was very popular many years ago, several countries have now stopped using them because staff (and patients) can become intolerant to latex after long exposure.

## Remove slip and trip hazards from the floor

If there are any pools or puddles of fluid on the floor then please mop it up immediately. This is to prevent someone from slipping and falling. If it is something acidic or a dangerous chemical please make sure you wear PPE and follow your local hazardous materials recommendations for clearing it up – this will prevent both injury to yourself (i.e. prevent a skin chemical burn) and damage to the flooring.

**‘Always look after your body when you are at work’**

Many hospitals have ‘wet floor’ signs that you can place on the wet floor area until it is cleaned up or it dries. This will help to prevent people from slipping in that area.

If you see any electrical cords or anything else on the floor, immediately secure it or tidy it away. This is to prevent someone from getting their feet caught and tripping over. The rule is: ***If you see it – clear it.*** Don't wait for someone else to do it. **You** do it.

## Use your body carefully

Always look after your body when you are at work. This means you must be careful when you are lifting or moving an object (including a patient) and/or doing repetitive movements. Here are some good tips you can apply:

- Always ask someone to help you move or lift an object that is heavy or difficult.
- Never twist your spine. Try and keep it straight.
- Push rather than pull.
- Try and keep heavier items at good body height level. i.e. not on a high shelf or low shelf.
- Use your stomach (core) and legs to lift and push up – avoid using your back.
- Get close to an object so it will make it easier to move something.
- Wear body braces (if available) such as lifting belts.

## Avoid repetition injury

This happens when you keep doing the same thing, in the same position, for extended periods of time; for example, people in an office sitting at a desk and typing. The key is to prevent these movements leading to strains, aches and, in some instances, severe pain. Here are some suggestions on how to prevent repetition injury:

- Move around between jobs.
- Alter your tasks or the way you do them.
- Change your environment.

## Prevent needle stick injury

Health care workers, and especially nurses, can sustain a needle stick injury if they do not practice safe needle-handling practices.

If you get a needle stick injury you need to immediately notify your manager and follow your hospital policy. If there is a risk of infection you may need prophylactic treatment – ask a doctor for advice. The best thing to do is to prevent it from happening in the first place.

Here are some recommendations.

- Never re-cap a needle.
- Never take a used needle from the hand of another person. Instead, ask the person to place the sharp item into a needle-container where it can be seen clearly.
- If you are the scrub nurse, never pass a needle or sharp blade to a surgeon when they are distracted, as it might harm them. Make sure you inform them that you are handing them the item so they can be alert and can safely take the item from you.
- Blades are to be handled with a special forceps that is strong enough to grasp the blade for placement onto and off the handle's shaft. Never use fingers.
- Only fill a sharps container to the fill line.
- Never grab or stick your hand inside any bowl or container without looking first. Sharp items (i.e. suture-need les) may have been accidentally left inside.

With thanks to the Fred Hollows Foundation New Zealand.
